# Supplementary material for: Preliminary Evaluation of Blending, Tuning, and Scaling Parameters in ssGBLUP for Genomic Prediction Accuracy in South African Holstein Cattle
Source: Animals (Basel). 2025 Sep 30;15(19):2866. doi: 10.3390/ani15192866 (PMC12523694; doi:10.3390/ani15192866)
Supplement: Supplementary file 1 [file animals-15-02866-s001.zip › animals-3738476-supplementary.pdf]

Table S1: Pearson correlation between EBVs and GEBVs from full (including all phenotypes) and reduced (excluding phenotypes of 390 animals) models for Holstein cattle, with mean individual accuracy shown in brackets.

Trait	EBV_{Full} [1]	$GEBV_{Full}$ [2]	$EBV_{Reduced}$ [3]	$GEBV_{Reduced}$ [4]
Milk (kg)	— (0.61)	0.87 (0.61)	0.28 (0.61)	0.26 (0.61)
Protein (kg)	— (0.58)	0.86 (0.58)	0.27 (0.58)	0.25 (0.58)
Fat (kg)	— (0.59)	0.88 (0.59)	0.27 (0.59)	0.25 (0.59)

[1] EBVs from full dataset using pedigree-based ABLUP

[2] GEBVs from full dataset using ssGBLUP

[3] EBVs from reduced dataset using ABLUP

[4] GEBVs from reduced dataset using ssGBLUP

Note: Mean individual accuracy values are shown in parentheses and were calculated using the formula:

$$\text{Accuracy} = \sqrt{1 - \frac{PEV}{\sigma_a^2}}$$

where PEV is the prediction error variance and σ_a^2 is the additive genetic variance.
